# Cas13d-mediated gene knockdown in CAR T cells: towards off-the-shelf cancer treatment

**DOI:** 10.1038/s41392-024-01830-3

**Published:** 2024-04-26

**Authors:** Midori Johnston, Nadine Urban, Can Dincer

**Affiliations:** 1 FIT Freiburg Centre for Interactive Materials and Bioinspired Technology, University of Freiburg, Freiburg, Germany; 2 IMTEK – Department of Microsystems Engineering, University of Freiburg, Freiburg, Germany

**Keywords:** Haematological diseases, Cancer therapy, Immunotherapy, Molecular biology

In a recent study published in *Cell*, Tieu et al. used RfxCas13d to dynamically enhance performance and longevity of chimeric antigen receptor (CAR) T cells by massively multiplexed gene knockdown and thereby, move the treatment one step closer towards “off-the-shelf” next-generation medicine.^1^

Surgery, chemotherapy, radiation, and targeted drug therapies are widely understood as the foundations of cancer treatment. Immunotherapy, sometimes referred to as the “fifth pillar,” usually describes either administration of drugs that will boost the patient’s endogenous immune system to shrink a tumor or immune checkpoint inhibitors.

In CAR T cell therapy, however, a patient’s own immune system is modified in order to disrupt the tumor cells’ immune evasion. Herein, a patient’s T cells are isolated, engineered and cultured to express a chimeric transmembrane receptor that will target a surface antigen, specific to the tumor cells. The goal of this treatment is targeting and destroying cancer cells after recognition by the CAR T cells upon their re-infusion into the patient’s bloodstream. The approach is predominantly used to combat hematologic forms of cancer like certain types of leukemia, lymphoma and multiple myeloma. But while there were remarkable results and, in some cases, even complete remission, the (side) effects, including mass die-off of antibody-producing B cells, neurologic toxicity, infections, T cell exhaustion, and cytokine release syndrome (CRS), should not be underestimated. And even though the treatment has entered the mainstream of next generation medicine, it is still inaccessible to the broader public, due to its price point at around $450,000.^2^

The development of methods that could move this extremely personalized approach towards off-the-shelf medication have the potential to drastically lower the costs and accelerate availability to the patient. One possibility is the collection and modification of T cells from healthy donors instead of the patient. Another, is the combination of CAR T cell therapy with transcription activator-like effector nucleases or clustered regularly interspaced palindromic repeats (CRISPR) technologies to induce the T cell’s production of CARs via knock-in of the desired genetic sequences. The problem with these, however, is the permanency of the genetic alterations made, as well as, with CRISPR-Cas9’s, potential unintended genotoxic side effects.^3^

**Fig. 1:**
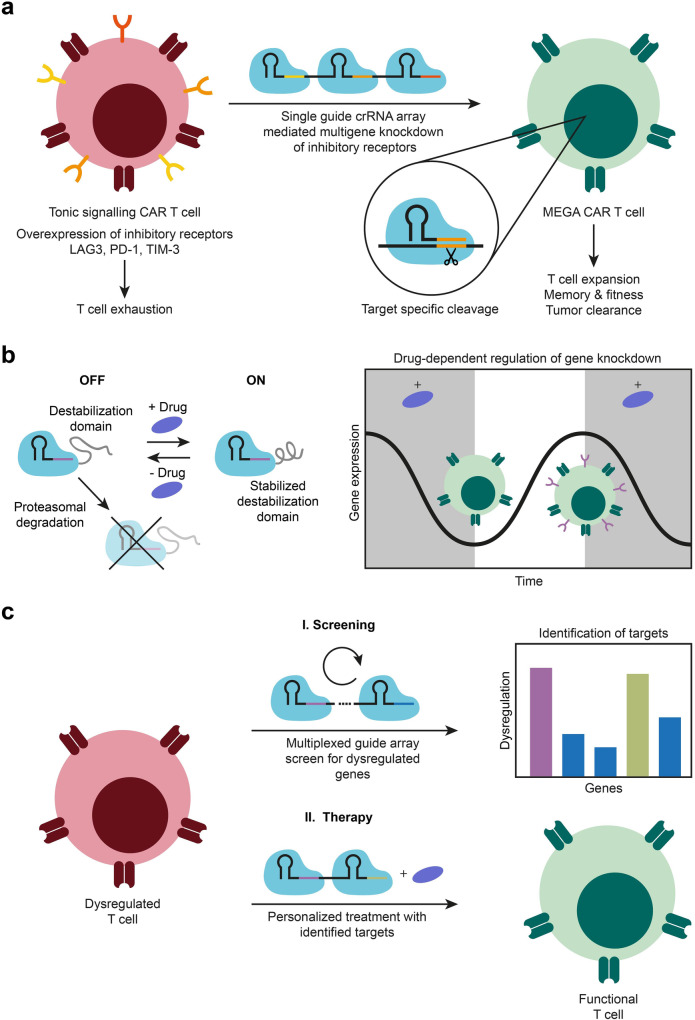
CRISPR/Cas13d mediated gene knockdown in T cells. **a** Reversion of tonic signaling phenotype in CAR T cells. The tonic signaling CAR T cell model overexpresses three inhibitory receptors (LAG3, PD-1, TIM-3) which lead to a T cell exhaustion phenotype. By transducing these cells with an array of guide crRNAs, the respective genes are knocked down by target specific Cas13d mediated RNA degradation. As a result, the exhaustion markers are no longer expressed, leading to improved T cell function such as cell expansion, memory, fitness and even tumor clearance. **b** Drug-inducible gene knockdown. A destabilization domain, fused to Cas13d leads to proteasomal degradation of the effector, disabling gene knockdown in the ground state. By addition of trimethoprim, the domain is stabilized and Cas13d cleavage is enabled. The system is reversible and can be utilized to dynamically regulate gene knockdown of T cell receptors by administration or withdrawal of the drug. **c** Potential MEGA-based T cell diagnostics. Dysregulated T cells could be extracted, transduced with the multiplexed guide array and screened for abnormally expressed genes. Afterwards, the cells could be transduced with guides targeting the identified genes, restoring physiological T cell function. Together with either inducible or permanent gene knockdown or in combination with other drugs, an individualized treatment plan could be implemented

Reporting in *Cell*, Tieu and colleagues recently introduced Multiplexed Effector Guide Arrays (MEGA) for dynamically regulated knockdown of multiple target genes on the transcriptome level in primary human T cells without affecting genomic DNA by employing RfxCas13d.^1^ This CRISPR effector is approximately two thirds the size of Cas9 and, unlike other Cas13a proteins, does not, or only to a minute extent, exhibit collateral trans-cleavage activity. In the context of this study, it was employed to revert T cell exhaustion in HA-28ζ CAR T cells, a well characterized model for tonic signaling, by knockdown of three inhibitory receptors, LAG3, PD-1, TIM-3 (Fig. 1a). To this end, primary human T cells were first transduced with RfxCas13d and HA-28ζ CAR, and then with a crRNA array, consisting of multiple sequentially positioned crRNA guides. Single, double and triple knockdowns of the respective target genes could be successfully performed while only minimal off-target effects were reported. Interestingly, the knockdown efficiency for some genes was influenced by the position of the guide sequence within the array. Therefore, when designing new arrays different positional permutations should be tested to achieve an optimal efficiency of the system. In order to gain temporal control of gene knockdown, RfxCas13d was fused to a destabilization domain that leads to proteasomal degradation in the steady state and can be stabilized with the antibiotic trimethoprim. Following this approach, the authors showed a reversible, drug-dependent expression of CD46 (Fig. 1b). MEGA was subsequently employed to study genes involved in purinergic signaling and glycolysis. Through knockdown of the identified genes, enhanced anti-tumor activity and improved cell fitness in dysfunctional CAR T cells could be induced. In addition, safety and efficacy of the treatment was improved by targeting proximal T cell activation signaling elements, enabling receptor independent regulation of CAR T cells.

Targeting T cell exhaustion, a major problem of CAR T cell therapy, MEGA’s potential was demonstrated by simultaneously suppressing the upregulation of three exhaustion markers and thereby improving longevity and tumor targeting.

In contrast to CRISPR/Cas9 systems, MEGA, employing the RNA recognizing and cleaving Cas13d, acts on the transcriptome instead of the genome level. It would be interesting to test a control element that also functions on the transcription level, i.e., inducible promoters: Regulation on the transcription level tends to be faster than at the protein level and is more energy and resource-efficient for the cell. It is also important to note that, neither cell culture, nor animal models provide a completely realistic prediction of the system’s actual performance and more precise control over CAR T behavior does not necessarily translate to improved safety and anti-tumor efficiency in cancer patients. Clinical trials are required in order to verify this effect.

Combining conventional or next-generation medicine with CRISPR-Cas13d could involve multiplexed genetic knockdown to profile key genes involved in pathogenesis, disease progression, the molecular signature of a specific disease, or, in the case of CAR T cell therapy, treat CRS (a potentially fatal side effect of the treatment). Therein lies great potential for diagnostic applications as well as tailoring personalized treatment options:^4^ potential drug targets, as well as drug sensitivity or resistance could be studied to predict or optimize the patient’s response to particular therapies (Fig. 1c). Possible fields of applications would be autoimmune diseases or allergies, where MEGA could offer a more precise and controlled alternative to traditional treatment.^5^ The proteins involved in the immune system’s overreaction could be temporarily downregulated at the transcription level to attenuate disproportionate reactions to non-pathogenic stimuli.

Overall, the cross-over of the two technologies creates a straightforward and fast method of transcriptional engineering for optimization of functional morphology. Its compact implementation and drug-inducible regulation hold massive potential for further improvements in safety, affordability and accessibility of the treatment. Having demonstrated its versatility in human T cells, MEGA could also be implemented for other cells or organisms. This study could pave the way for gaining further insights into the physiology of disease models, offering personalized and rapidly adjustable therapy options.
